# Maxillary defect correction by aesthetics

**DOI:** 10.11604/pamj.2021.39.160.30163

**Published:** 2021-07-01

**Authors:** Rajiv Dharampal Bhola, Waqar Mohsin Naqvi

**Affiliations:** 1 Department of Prosthodontics, Sharad Pawar Dental College, Datta Meghe Institute of Medical Sciences, Sawangi (M), Wardha, India,; 2 Department of Community Health Physiotherapy, Ravi Nair Physiotherapy College, Datta Meghe Institute of Medical Sciences, Sawangi (M), Wardha, India

**Keywords:** Prosthodontics, mandibular defect, dental prosthesis

## Image in medicine

A young patient of age 34 years complains of poor aesthetic because of maxillary defect. The patient complains of difficulty in speech, swallowing, mastication and cosmetic disfigurement which was the main concern for which patient reported to the outpatient department (OPD) where examination was done. This revealed absence of incisors and anterior mandibular and anterior maxillary defect and as mentioned by patient poor cosmetics was the main concern, pre-examination was done and surgery was conducted. Consent was taken and case history was recorded. An implant supported Prosthesis was planned for maxilla and mandible in anterior region. Aesthetic rehabilitation was satisfactorily achieved. The (A) depicting pre-operative image of the patient with missing incisors and (B) shows the defect was corrected by prosthesis.

**Figure 1:**
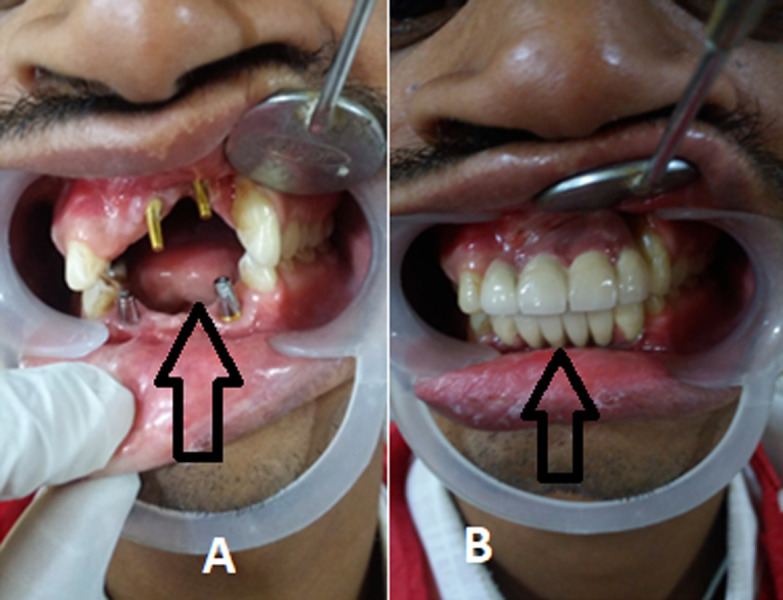
A) showing pre-operative image of the patient; B) correction done by implant supported bridge

